# Risk Tools Predict Recidivism for Men With Low Intellectual Ability and a History of Sexual Offending

**DOI:** 10.1177/10790632261429124

**Published:** 2026-03-02

**Authors:** R. Karl Hanson, Kelly M. Babchishin, Kelsey May, Benjamin Reid, Robin J. Wilson

**Affiliations:** 1 Department of Psychology, 6339 Carleton University, Ottawa, ON, Canada; 2 The Society for the Advancement of Actuarial Risk Needs Assessment (SAARNA), Kingston, ON, Canada; 3 Department of Psychiatry and Behavioural Neurosciences, McMaster University, Hamilton, ON, Canada

**Keywords:** risk assessment, low intellectual ability, intellectual development disorder, meta-analysis, sexual offending

## Abstract

Standardized risk assessments are essential to evidence-based responses to criminal behaviour, including sexual offending. Since the 1990s, many actuarial and structured risk assessment instruments have been developed that are now routinely used in sentencing, treatment, and post-release risk management. The majority of these scales, however, were developed using undifferentiated groups, raising questions about their utility within meaningful subgroups, such as persons with low intellectual ability. This study presents meta-analytic findings of the predictive accuracy (discrimination) of risk tools for crime and violence when used with men with low intellectual ability and a history of sexual offending. We also examined age as a recidivism risk predictor. Database searches identified 15 distinct samples (N = 923). Age, as a single variable, showed moderate to large effects for sexual, violent, and general (any) recidivism. Overall, the predictive accuracies of the instruments were moderate and similar to those observed for other samples. Amongst the measures investigated, a measure specifically for persons with low intellectual ability (ARMIDILO-S; Boer et al., 2013) demonstrated the highest predictive accuracy. Larger effects were found when recidivism was measured by staff reports than by official records (e.g., charges, convictions). Our findings support the use of existing risk tools with men with low intellectual ability. Researchers should privilege staff reports over official records of recidivism for studies concerned with sexual recidivism.

## Introduction

Effective correctional interventions require risk assessment. According to Andrews et al.’s (1990; Bonta & Andrews, 2024) Risk/Need/Responsivity (R/N/R) model, risk tools inform how much intervention is needed (the Risk Principle) and identify factors to be addressed in supervision and treatment (the Need Principle). Once risk and need have been established, how best to intervene requires attention to often-unique attributes of those individuals (e.g., intellectual ability, learning style, motivation), in order to maximize intervention effects (the Responsivity Principle – see Wilson & Yates, 2009). Treatment programs that align with the R/N/R principles are those most likely to reduce sexual recidivism (Hanson et al., 2009; Holper et al., 2024). Risk tools also inform the implementation of sex-crime public protection measures, such as civil commitment and sexual offender registries.

There is considerable research on risk tools for persons with a history of sexual offending (Hanson & Morton-Bourgon, 2009; van den Berg et al., 2018) and they are widely used in practice (Bourgon et al., 2018; Kelley et al., 2020). Given that risk tools are developed on mixed groups, there is an ongoing need for validation studies on subgroups. Not all differences justify subgroup analyses. In the context of recidivism risk assessment, meaningful differences would include subgroups with distinctive offending patterns, risk factors, opportunities and constraints on offending (e.g., family/living arrangements), as well as distinctive expectations of – and treatment by – the criminal justice system. One such subgroup are men with low intellectual ability and a history of sexual offending.

As used in this paper, low intellectual ability refers to functional impairments resulting from difficulty understanding and resolving cognitively demanding life challenges (American Association on Intellectual and Developmental Disabilities, 2010; American Psychiatric Association, 2022). Persons with low intellectual ability may be diagnosed with Intellectual Developmental Disorder (DSM-5-TR; American Psychiatric Association, 2022) or a disorder of intellectual development (International Classification of Diseases – 11; World Health Organization, 2025). According to DSM-5-TR, the threshold for low intelligence is usually set at an IQ of 65 to 75 (approximately two standard deviations below the mean, lowest 1% to 2% of the population). Current practice, however, tends to emphasize adaptive functioning over numerical IQ scores (Mela et al., 2025). Although there are degrees of intellectual disability (e.g., mild, moderate, severe, profound), most cases are mild, particularly cases that proceed to the criminal justice system. Transgressions by people with very serious cognitive impairment are usually directly managed by health and social services, without recourse to police and courts (Taylor & Lindsay, 2018). Consequently, we use the term low intellectual ability to indicate clinically significant low intellectual ability, which may or may not meet formal diagnostic criteria for DSM-5-TR or ICD-11 diagnoses. As noted below (Studies Included), most of the sexual recidivism studies of men with cognitive deficits predominantly included men whose cognitive deficits were classified as “borderline” (i.e., low intellectual ability was a clinical concern but insufficient for a formal diagnosis).

People with low intellectual ability are disproportionally represented among people convicted of crimes (Lunsky et al., 2024), including sexual offences (Hawk et al., 1993). Between 4% and 14% of incarcerated individuals in the United States have been diagnosed with an intellectual developmental disorder (Salekin et al., 2010), which is substantially higher than the base rate of 1% to 2% in the general male population (McKenzie et al., 2016). In a population study of people receiving forensic assessments in Sweden, persons with disorders of intellectual development were twice as likely to have a previous sex crime (10.8%), or an index sex crime (26.2%) compared to persons without such disorders (5.2% and 11.5% respectively; Edberg et al., 2022).

The reasons for the overrepresentation of persons with low intellectual ability in official criminal records are not fully known. Contributing factors could include prejudice and bias, and the inability to avoid detection. It could also be related to distinctive forms of sexual transgressions committed by people with low intellectual ability. Hingsburger et al. (1991) used the concept of “counterfeit deviance” to describe how the restrictive living conditions of many persons with low intellectual ability can transform normally appropriate behaviour (e.g., masturbation) into sexual transgressions (e.g., masturbating in public). Whereas public masturbation is a reliable indicator of paraphilia in many populations, it could simply indicate poor decision-making concerning location and timing for those living in crowded, supervised housing that lack the privacy that persons without disabilities take for granted (Wilson et al., 2014). The extent to which counterfeit deviance actually contributes to sexual offending among men with low intellectual ability remains an open question (Griffiths et al., 2013).

Poor executive functioning and low self-control are reliable indicators of offending in general (Gottfredson & Hirschi, 1990) and sexual offending in particular (Mann et al., 2010; Seto et al., 2023). In the general population, executive functioning reliably increases with age, with consolidation in the early 20s (Reynolds & MacNeill Horton, 2008). The age-crime curve largely follows the development of executive functioning and decreases in associated impulsive, poorly considered behaviours (e.g., Hirschi & Gottfredson, 1983; Sampson & Laub, 1993). The incidence of sexual crime is highest in the early teenage years (13–15) and declines thereafter, with a second smaller peak in the 30s (Kong et al., 2003). This suggests that a substantial proportion of sexual offending is related to immature sexual and general self-regulation. If sexual offending by people with low intellectual ability is related to delays in the development of sexual self-regulation, this would increase the number of years at which they are at risk for poor sexual and general decision-making, sometimes rising to the level of charged sexual offending.

Although the relationship between age and sexual recidivism is well established in mixed groups of men (Hanson & Bussière, 1998; Helmus, Thornton, et al., 2012; Thornton, 2006), less is known about the relationship for men with low intellectual ability. Given the protective effect of advanced age on all types of crime, age should also be related to reduced sexual crime among people with low intellectual ability. Whether it is more or less important is an open question. It is possible that deficits in cognitive development could decrease the protective effect of age. Conversely, aging during adulthood could be more protective: If the developmental disorder involves a delay (not just a lower ceiling), then their executive functioning could show greater relative improvement during the adult years than among men with average intellectual ability, who would have largely completed this aspect of brain development by their early to mid 20s (Best & Miller, 2010, for a review). In the one study we identified that directly compared the predictive accuracy of age on violent and sexual recidivism, age was a stronger predictor for individuals without intellectual disability than for those with intellectual disability (Edberg et al., 2022).

Just as the effect of age could vary across intellectual ability, so too could many of the factors considered in recidivism risk tools. Compared to people without intellectual disabilities, those with low intellectual ability are less likely to be married, less likely to be employed, and more likely to be in supervised housing (Charpentier & Carter, 2023). Official criminal justice interventions, which are reliable recidivism predictors in mixed samples, may have less importance with for persons with low intellectual ability because of diversion and the availability of unofficial sanctions within already highly controlled settings (e.g., group homes, state-run hospitals). Some standard items on risk tool may increase their scores without the corresponding increase in risk for recidivism (e.g., Craig & Hutchinson, 2005). For example, compared to persons of average intellectual ability, men with low intellectual ability are unlikely to have a history of stable, long-term cohabitation with an intimate partner (items on commonly used risk tool, such as Static-99R [Helmus, Thornton, et al., 2012] and STABLE-2007 [Hanson et al., 2015]), more likely to have males as victims (an item on several risk tools) because persons in care settings are typically housed by gender, and are more likely to demonstrate emotional identification with children or childhood (STABLE-2007 item) because their intellectual functioning is comparable to that of much younger persons.

A previous meta-analysis by Hanson and colleagues (2013) found moderate to large effect sizes for sexual recidivism (Cohen’s *d*) among men with development delays for some commonly used risk tools (Rapid Risk Assessment for Sexual Offense Recidivism [RRASOR], Hanson, 1997; Static-99, Hanson & Thornton, 2000; Static-99R, Helmus, Thornton, et al., 2012). Their results, however, were based on only two to four studies, with small sample sizes. The results of subsequent studies have been variable. For example, Stephens et al. (2018) found low to moderate predictive accuracy for Static-99R (sexual charges; *Harrell’s C* = 0.60) in a sample of 78 men with IQ less than 80. In contrast, Callahan et al. (2024) found no relationship between Static-99R scores and the likelihood of being reincarcerated for a new sexual offence among 213 men with borderline or mild intellectual disability in New Jersey (*AUC* = 0.51). A risk tool specifically designed for the men with low intellectual ability (Assessment of Risk and Manageability for Individuals with Developmental and Intellectual Limitations who Offend Sexually [ARMIDILO-S]; Boer et al., 2013) showed good predictive accuracy (discrimination) for sexual recidivism (*AUC* = 0.92, Lofthouse et al., 2013; *AUC* of 0.70 to 0.90, Pouls & Jeandarme, 2023), as did the Violence Risk Appraisal Guide (VRAG; Quinsey et al., 1998), an actuarial risk tool designed for general violence (*AUC* = 0.74, Fedoroff et al., 2016). One potential benefit of the ARMIDILO-S is that it not only addresses characteristics of the individual, but also the risk and protective contributions of the staff and the person’s living environment.

It is difficult to tell how much of the variation across studies is due to differences in risk tools, outcome measures, or other design characteristics. Even when the total sample size was not small, the statistical precision of the estimates would be low because of the absolute number of recidivists in any particular study was small (typically less than 20). Meta-analysis is the accepted method of aggregating the results of small, underpowered studies (Borenstein et al., 2021). Not only does meta-analysis provide better estimates of effect sizes than individual studies, meta-analyses also examine the extent to which variation across samples and settings is more than expected by chance (i.e., sampling error).

### Current Study

We conducted a meta-analysis of the predictive accuracy (discrimination) of recidivism risk tools among men with low intellectual ability and a history of sexual offending. We also examined age as a recidivism risk indicator (single variable). Effect sizes were aggregated using fixed-effect and random-effects models. Four types of recidivism were examined: sexual, non-sexual violent, violent, and general (any; i.e., sexual, nonsexual violent, and nonviolent offences). Our analyses were based on the classification of offences (e.g., sexual, violent, non-violent) used by the authors of the original studies. For violent offences, four studies excluded non-contact sexual offences, two studies included all sexual offences as violent, two studies did not specify the type of sexual offences included, and the remaining seven studies did not analyze an overall category of violence. We only considered the ability of risk tools to differentiate between recidivists and non-recidivists. Investigating the calibration of these risk tools was not within the scope of the current study.

We expected similar results as have been found for other mixed samples: (a) overall, recidivism risk tools would show moderate predictive accuracy (discrimination); (b) risk tools designed for sexual recidivism would do better for sexual recidivism than risk tools designed for general (any) or violent recidivism; (c) risk tools designed for general or violent recidivism would do better for those outcomes than risk tools designed for sexual recidivism; (d) there would be little difference between the general and violent risk tools because of the substantial overlap in the risk factors for crime in general and violent crime, in particular; and (e) young persons would be more likely to reoffend than older persons. Given the encouraging results of the ARMIDILO-S (Boer et al., 2013), we were cautiously optimistic that risk tools specifically designed for persons with low intellectual ability populations would perform better than risk tools designed for general (mixed) populations.

## Method

### Inclusion Criteria

Prospective or pseudo-prospective studies were eligible for inclusion. Pseudo-prospective studies have the same design features as truly prospective studies (i.e., assessment prior to recidivism being known, definable follow-up period, dichotomous outcome) but use existing data instead of waiting for the outcome to occur at a future date. The sample had to be comprised of men aged 18 or older with a sexual offence history and sufficiently low intellectual ability that it was a clinical concern (including those with borderline intellectual functioning), as determined by specialized testing, institutional placement, and/or staff ratings (see below). Recidivism was defined by staff report or official criminal justice records (conviction, charge), and classifiable as sexual, non-violent, violent, or any (general) recidivism. Studies must have included at least 10 participants. See the coding manual (available at Open Science Framework [OSF] link: https://osf.io/7vr43/overview) for a more detailed description of the inclusion and exclusion criteria.

### Search Strategy

**Figure 1.**
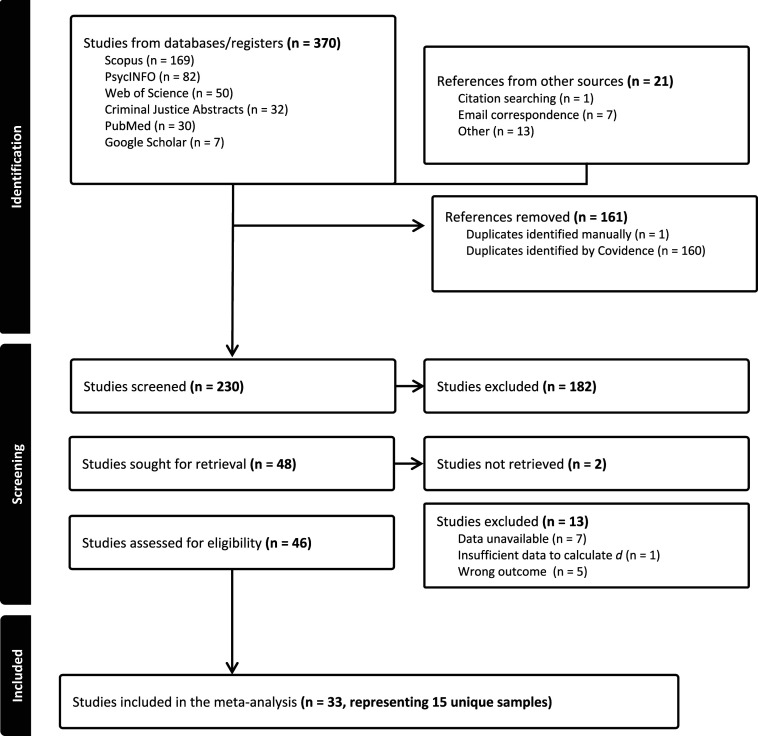
Study selection flowchart

The following databases were searched in the fall of 2024: Scopus, PsycINFO, Web of Science, Criminal Justice Abstracts, PubMed, and Google Scholar. For each of the databases listed above, the following search terms were used: “sex offen*”, or “sexual abuse”, “intellectual disability”, or “intellectually disabled”, or “developmental disability”, or “learning disability”, “mental retard*” and “recid*” or “reoffen*”. Results were then uploaded to Covidence, which is a web-based software program that assists in the systematic review process, including study screening and data extraction (Veritas Health Innovation, 2024). Duplicate entries were removed, and reference lists of relevant studies were reviewed for additional studies that met the inclusion criteria. Authors were then contacted for additional data if the original study did not have sufficient information required for coding. The search terms listed above yielded 394 initial results that required sorting. Of those 394 results, only 15 unique samples met the inclusion criteria for the meta-analysis. The study selection procedure is presented in Figure 1. We re-ran the search in August 2025 to identify any additional studies: none were found.

### Studies Included

Table 1 provides a brief description of the 15 studies in this meta-analysis, and the Study ID legend is in the Appendix. For ten studies, additional data (beyond what was presented in the reports) was provided by the authors. The additional data included updated recidivism information, and the statistics needed to calculate the effect sizes. The sample sizes per study were relatively small, with a median of 44 (mean of 62.3, SD = 62.9, range from 11 to 271, total sample of 934). Most studies were published (13/15; 87%) and were produced between 2001 and 2024 (median of 2016). Most of the samples were from treatment programs (*k* = 9) or selected to be high-risk/high-need (*k* = 4), with only two being from routine correctional samples. Of the studies that reported Static-99R scores (see Risk Tools below), the average scores for four of the five samples were in the Above Average Risk category (Level IVa), and the other sample was Average (Level III). The most common offence type within each study were sexual offences against children (*k* = 11), particularly unrelated children (*k* = 8). One study (Baudin et al., 2021) predominantly included men who had sexually offended against adults. Three studies did not report the predominant sexual offence types.

**Table 1.** Studies Included in the Meta-Analysis

Study	Country	Recidivism information	Setting	Sample size^a^	Sexual Recidivism Rate^b^	Average Follow-up (Months)	Proportion borderline	Additional data provided
Baudin et al., 2021	Sweden	Official	Institution	11	45.5	180	36.4^c^	Yes
Blacker et al. (2011)	U. K.	Staff	Community	44	45.5	109	9.1	No
Callahan et al. (2024)	U. S.	Official	Institution	271	3.7	69	100	No
Delforterie et al. (2023)	Netherlands	Official	Institution	63	7.9	81	0	Yes
Federoff et al., 2016	Canada	Staff	Community	51	45.9	32	0	Yes
Hanson et al. (2015)	Canada	Official	Community	38	21.1	89	2.6	Yes
Hanson et al. (2024)	Canada	Official	Community	39	43.6	238	74.4	Yes
Lofthouse et al. (2013)	U. K.	Staff	Community	64	32.8	72	34.4	No
McGrath et al. (2012)	U. S.	Official	Community	18	16.7	60	100	Yes
Pouls and Jeandarme (2023)	Belgium	Staff	Institution	38	10.5	11	31.6	Yes
Sindall (2012)	U. K.	Staff	Combined	16	31.3	5	100	No
Sowden and Olver (2017)	Canada	Official	Institution	95	21.1	150	0	Yes
Stephens et al. (2018)	Canada	Official	Combined	78	19.2	125	65.4	Yes
Tough (2001)	Canada	Staff	Community	81	17.3	85	25.9	Yes
Wilcox et al. (2009)	U. K.	Official	Community	27	25.9	76	100	No

*Note.* Official recidivism included arrests/charges and convictions.

^a^The largest sample for any predictor of any recidivism outcome (sexual, violent, general).

^b^The sexual recidivism rate for the largest sample, which could be smaller than the total sample reported in the previous column.

^c^Based on *N* = 13.

The determination of clinically significant low intellectual ability (i.e., intellectual deficiency, disorder of intellectual developmental) was based on standardized testing (*k* = 5), or combination of testing and functional impairment (*k =* 7 studies); three studies did not describe their procedure for determining low intellectual ability. Six of the studies reported that most of their cases had borderline intellectual functioning (75 < IQ <85; *k* = 6). The proportion of mild, moderate, and severe cases was rarely reported in the original studies and not coded for this meta-analysis study. We expect, however, that the use of these risk tools with persons with moderate or severe disability would be rare.

The average follow-up time was 7.1 years (median = 6.7; SD = 4.8, range from five months to 19.8 years). The overall sexual recidivism base rate was 19.5% (171/876, *k* = 15; 28.9% [81/280; *k* = 6] based on staff reports and 15.1% [90/596; *k* = 9] based on official reports).

### Procedure

Studies were independently coded by the two trained undergraduate students (KM and BR) using a standardized coding manual, after two practice studies. Rater reliability was conducted on a total of 54 variables coded across 13 samples using either the intraclass correlation coefficient (ICC, for ordinal/linear variables) or Cohen’s kappa (for dichotomous or categorical variables; Hanson, 2022). The ICC was calculated using the two-way mixed effects model with absolute agreement and reported for a single measure. The ICC values ranged from .71 to 1.0, with a median of 1.0 (22 variables). Both ICC and Cohen’s kappa assess interrater reliability while accounting for chance agreement (Fleiss et al., 2003; Chapter 18). Kappa values ranged from .44 (86.6% agreement) to 1.0 (100% agreement), with a median of 1.0 (100% agreement; 32 variables). There was perfect agreement (ICC = 1.0) on the 280 effect sizes identified in this study. This unusually high agreement could be attributed the coders mostly working directly from the data provided by the authors of the original studies.

### Plan of Analysis

The authors take responsibility for the integrity of the data, the accuracy of the data analyses, and have made every effort to avoid inflating statistically significant results. Cohen’s *d* was used for the effect size, which was calculated from the means and standard deviations in 13 studies (authors provided the raw data for 9 of the studies while for the remaining 4 studies this information was in their papers). The Area Under the Curve (AUC) from receiver operating characteristic (ROC) analyses was used solely for 2 studies, and in combination with means and standard deviations (*k* = 1). The AUC from ROC was transformed into a Cohen’s *d* value using the mathematic approach found in Rice and Harris (2005). A Cohen’s *d* value of ±0.20 is a small effect, ±0.50 is a medium effect and scores of ±0.80 or higher are a large effect (Cohen, 1988), and a *d* of ±0.15 is defined as meaningful for individual risk factor (Mann et al., 2010). A positive Cohen’s *d* value indicates that recidivists had a higher mean on their risk score measures or were younger than the non-recidivists.

Fixed- and random-effects analyses were run independently by two analysts using either IBM SPSS Statistics (Version 29.0.2.0; IBM Corporation, 2023) or R (version 4.4.2, R Core Team, 2017; ‘metafor’; Viechtbauer, 2010). There had to be three or more samples to contribute to each analysis. Both statistical programs produced equivalent results within rounding error, with the exception of the prediction intervals (see below). The fixed-effect analysis assumes that the samples were drawn from the same population; consequently, any observed variability would be due to sampling error. Random-effects analysis assumes that samples come from different populations, therefore factoring in variability between populations in the analysis (*T*^*2*^). The *Q*-statistic for the fixed-effect analysis is important as a low value that is not statistically significant indicates that the variability between studies can be explained due to chance (i.e., not more than would be expected by chance). *I*^2^ is calculated from the Q-statistic to describe the variance found in the effect sizes due to true differences rather than sampling error (Borenstein et al., 2021, pp. 139–150). Potential outliers were identified and excluded if the following three criteria were met: the *Q* (variability) was significant (*p* < .05), the effect size was an extreme value (i.e., highest or lowest effect size), and the *Q* (variability) was reduced by more than 50% when the sample was removed (Hanson, 2022, pp. 265–266). Analyses were conducted again with the remaining studies. Results are reported with and without outliers.

Random-effects prediction intervals estimate the range in which the effect sizes of future studies are expected to fall, accounting for the variability across all studies used in the meta-analysis (Borenstein et al., 2021, pp. 119–125). The prediction intervals were calculated as *M* ± *Z*_α_ (*T*^2^ + *V*)^1/2^ (the default in *metafor*) rather than *M* ± *t*_α_ (*T*^2^ + *V*)^1/2^ (the default in SPSS) in order to maintain the same metric as the confidence intervals. In this formula, *M* represents the overall mean effect size, Z_α_ is the critical value from the standard normal distribution corresponding to the desired level of confidence (e.g., 1.96 for 95% confidence), *T*^*2*^ denotes the sample estimate of the variance of the true effect sizes (i.e., the amount of between-study heterogeneity), and *V* represents the sampling variance of the mean effect size from the random effects model. The use of *Z*_*α*_ (*metafor*) instead of *t*_*α*_ (SPSS) ensures consistency with the confidence interval metric, as *Z* is used in the construction of confidence intervals in both *metafor* and SPSS.

Post hoc moderator analyses explored the extent to which the effect size varied based on whether recidivism was defined by staff reports or by official records (charges, convictions).

The dataset (in IBM/SPSS. sav and EXCEL. csv) and associated syntax files (SPSS.sps and WORD files for R) are available on the website of the Open Science Framework (OSF): htttps://osf.io/7vr43/overview. The study was not pre-registered.

### Predictor Variables

#### Age

Age was coded as either the age at time of assessment or age when first at-risk after the index sexual offence (as per Static-99 R/Static-2002R coding rules). The average age of the samples ranged from 32 to 44 years (median of means: 36 years). All cases were adults (18+ years old).

#### Risk Tools

A risk tool was defined as a structured method of assessing the likelihood of recidivism based on factors specified in advance. We placed no restrictions on the method of combining the factors into an overall evaluation; however, all studies used a mechanical method of computing total scores (i.e., *actuarial* or *mechanical* risk tools using Hanson and Morton-Bourgon’s [2009] typology). Although some of the measures were designed to support risk level assignment by structured professional judgement (SPJ), all eligible studies reported only total scores for these measures. The following risk tools were examined in at least three studies.

##### Actuarial Risk Tools

**Rapid Risk Assessment for Sexual Offence Recidivism (RRASOR**; Hanson, 1997). The RRASOR is an actuarial risk assessment tool designed to assess the likelihood of sexual recidivism of adult males. It contains four items: prior sex offences, age younger than 25 years old, any male victims, and no prior relationship with the victim. Prior sex offences are coded on a scale of 0-3, where more convictions and charges result in a higher score. The total RRASOR score ranges from 0-6, with a higher score indicating that sexual recidivism is more likely to occur. Although it has demonstrated moderate predictive accuracy for sexual recidivism (Babchishin et al., 2012), the author no longer supports its applied use (Thornton & Hanson, 2016).

**Static-99** (Hanson & Thornton, 2000). Static-99 is an actuarial risk assessment tool designed to assess the likelihood of sexual recidivism in adult males. It contains 10 static items, two of which pertain to the age and relationship history. The remaining items are related to criminal history (sexual and violent) and victim characteristics. The total score ranges from 0–12, with the following risk-level labels: low (0–1), moderate-low (2–3), moderate-high (4–5), and high (6–12). Although Static-99 has moderate predictive accuracy (Helmus, Thornton, et al., 2012) and is still used in some jurisdictions, the authors recommend the revised version (Static-99R; Thornton & Hanson, 2016).

**Static-99R** (Helmus, Thornton, et al., 2012). The Static-99R is an updated version of the Static-99 with revised age weights, resulting in a total score range of −3 to 12 with the following risk categories: very low (−3 to −2), below average (−1 to 0), average (1-3), above average (4-5) and well above average (6+; Helmus et al., 2021). Static-99 R has high interrater agreement and moderate predictive accuracy. It is by far the mostly commonly used and most extensively researched sexual recidivism risk tool in the world (Helmus et al., 2022).

**Static-2002R** (Helmus, Thornton, et al., 2012). Static-2002R is another actuarial risk assessment tool for estimating the likelihood of sexual recidivism among adult males (Helmus et al., 2021). It has 14 items grouped into the following five domains: age at release, persistence of sexual offending, sexual deviance, characteristics of victims, and general criminality. Total scores range from −2 to 13, with the following risk level labels: very low (−2 to −1), below average (0 to 1), average (2–4), above average (5–6) and well above average (7+). Static-2002R has moderate predictive accuracy, similar to Static-99R (Babchishin et al., 2012).

**Violence Risk Appraisal Guide (VRAG;** Quinsey et al., 1998). The VRAG is an actuarial risk assessment tool designed to assess the likelihood of violent recidivism. It contains the following 12 items: living with both parents until age 16, early school maladjustment, history of alcohol problems, marital status, criminal history, failure on prior conditional release, age, any female victim, extent of victim injuries, Psychopathy Checklist – Revised (PCL-R; Hare, 2003) total score, DSM-III schizophrenia diagnosis, and personality disorder diagnosis meeting DSM-III criteria. The total scores range from −26 to 38, grouped into nine approximately equally populated categories. It has demonstrated a large AUC value for violent recidivism (Harris et al., 2015). Although it has been widely used, it is not currently recommended because it has been superseded by a revised version (Violence Risk Appraisal Guide – Revised [VRAG-R]; Harris et al., 2015; Helmus & Quinsey, 2020). The revised version removed items that were difficult to code (e.g., psychiatric diagnoses), revised the scoring of some items, and added four new items, resulting in a 12-item measure applicable to persons with a history of violent or sexual offending.

##### Mechanical Risk Tools

**Assessment of Risk and Manageability of Individuals with Developmental and Intellectual Limitations who Offend – Sexually (ARMIDILO-S**; Boer et al., 2013). The ARMIDILO-S is a type of structured risk assessment tool designed specifically for those with an intellectual or developmental disability who have a sexual offence history or exhibit inappropriate sexual behaviours. The ARMIDILO-S consists of dynamic risk factors, organized into stable factors (slow-changing factors), and acute factors (rapidly changing factors). Each factor (e.g., Item #7: Relationships) has a risk rating (e.g., unable to form bonds) and a protective rating (e.g., has caring relationships with non-family). It is recommended that the stable factors are scored about once a year, whereas the acute factors should be scored every two to three months. The stable and acute factors not only describe the person assessed (e.g., intimacy deficits, compliance with treatment), they also describe their support persons (e.g., communication among support persons, consistency of supervision/intervention) and aspects of the individuals’ housing arrangements (e.g., relationships with peers and roommates). The individual items are scored *No, Somewhat*, and *Yes* – for both “risk” or “protective” considerations. Evaluators are encouraged to use the ratings to develop a treatment-oriented case-formulation and provide an overall professional judgement of risk level (Low, Moderate, High) informed by the ARMIDILO-S Risk and Protective ratings along with a static, actuarial risk tool, such as Static-99 or RRASOR. For research purposes, however, the ARMIDILO-R stable and acute ratings were transformed into a three-point scale (0 = *No*, 1 = *Somewhat*, 2 = *Yes*, for risk ratings; 2 = *No*, 1 = *Somewhat*, 0 = *Yes*, for protective ratings), and summed into a total score that included both risk and protective ratings.

##### Other Risk Tools

Fifteen other risk tools were examined in one or two studies and contributed to the summary analyses but not analyzed separately. These included tools for general and violence risk (actuarial: General Statistical Information on Recidivism [GSIR; Nuffield, 1982], Sex Offender Risk Appraisal Guide [SORAG, Rice & Harris, 1997], Brief Assessment of Recidivism Risk [BARR-2002R, Babchishin et al., 2024], Risk Matrix-2000V [RM-2000V, Thornton, Fernandez, & Helmus, 2024]; SPJ: Dynamic Risk Outcome Scales [DROS; Drieschner & Hesper, 2008], HKT-30 [Werkgroep Risicotaxatie Forensische Psychiatrie. 2002]; mechanical: Psychopathy Checklist – Revised [PCL-R, Hare, 2003], Psychopathy Checklist – Screening Version [PCL-SV, Hart et al., 1995]), and risk tools designed specifically for sexual recidivism (actuarial: Minnesota Sex Offender Screening Tool – Revised [MnSOST-R; Epperson et al., 1999], Static-2002 [Hanson & Thornton, 2003; Phenix et al., 2008], Vermont Assessment of Sex Offender Risk – 2 [VASOR-2; McGrath et al., 2014], Violence Risk Scale – Sexual Offender version [VRS-SO; Olver et al., 2007]; mechanical: STABLE-2000, STABLE-2007 [Hanson et al., 2015], Sexual Violence Risk – 20 [SVR-20; Boer et al., 1997]).

## Results

**Figure 2.**
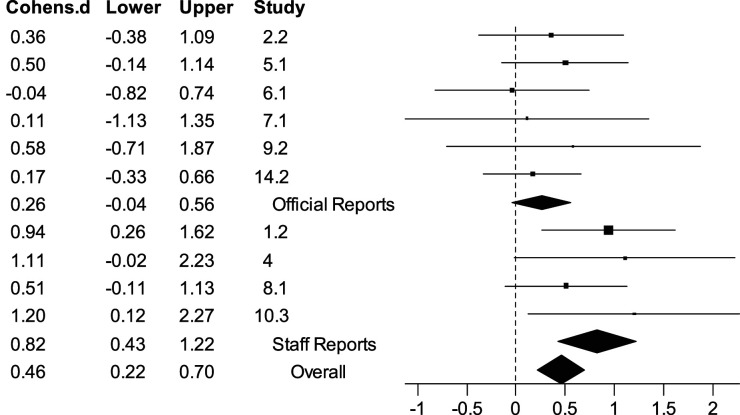
Fixed-Effect Forest Plot for the Relationship Between Age and Sexual Recidivism. *Note.* Diamonds indicate fixed-effect weighted averages for official reports (*k* = 6), staff reports (*k* = 4), and overall (*k* = 10)

As a single variable, age had a small to moderate effect for sexual recidivism (*d* = 0.46), a moderate effect for any violent recidivism (*d* = 0.66/0.63), and a large effect for any recidivism (*d* = 0.76; see Tables 2 and 3). The fixed-effect and random-effects analyses provided equivalent results because there was little between-study variability. For all recidivism outcomes, the variability did not exceed what would be expected by chance (all *Q* statistics were nonsignificant). There was, nevertheless, considerable range in the observed effects sizes (and large prediction intervals) because the sample sizes were small. As can be seen in the forest plot for age as a predictor of sexual recidivism (Figure 2), the effect sizes ranged from *d* = –0.04 to 1.20.

**Table 2.** Prediction of Sexual Recidivism for Men With Low Intellectual Ability and Sexual Offence Histories

Predictors	Fixed			Random		*k* (rec/*N*)	Studies
	*d* [95% CI]	*Q*	*I* ^*2*^	*d* [95% CI]	*PI*		
Age	**0.46 [0.22, 0.70]**	8.36 (*p* = .50)	0.0	**0.46 [0.22, 0.70]**	[0.22, 0.71]	10 (98/438)	1.1, 2.2, 4, 5.1, 6.1, 7.1, 8.2, 9.1, 10.1, 14.1
Actuarial for general recidivism	**0.39 [0.14, 0.63]**	3.61 (*p* = .61)	0.0	**0.39 [0.14, 0.63]**	[0.14, 0.63]	6 (103/311)	1.1, 5.1, 6.1, 11, 12, 14.1
Any VRAG/R	**0.42 [0.073, 0.77]**	1.14 (*p* = .57)	0.0	**0.42 [0.073, 0.77]**	[0.073, 0.77]	3 (55/139)	1.1, 5.1, 12
VRAG	**0.46 [0.11, 0.81]**	1.05 (*p* = .59)	0.0	**0.46 [0.11, 0.81]**	[0.11, 0.81]	3 (55/139)	1.1, 5.1, 12
Actuarial for sexual recidivism	**0.54 [0.35, 0.73]**	11.04 (*p* = .53)	0.0	**0.54 [0.33, 0.74]**	[0.25, 0.84]	13 (148/775)	2.1, 3, 4, 5.1, 6.1, 7.1, 8.1, 9.1, 10.1, 11, 12, 13.1, 14.1
Any static-99/R	**0.57 [0.36, 0.77]**	7.53 (*p* = .75)	0.0	**0.57 [0.36, 0.77]**	[0.36, 0.77]	12 (128/724)	2.1, 3, 4, 5.1, 6.1, 7.1, 8.1, 9.1, 10.1, 12, 13.1, 14.1,
Static-99R	**0.49 [0.24, 0.74]**	5.15 (*p* = .74)	0.0	**0.49 [0.24, 0.74]**	[0.24, 0.74]	9 (85/545)	2.1, 4, 5.1, 6.1, 7.1, 9.1, 10.1, 13.1, 14.1
Static-99	**0.66 [0.36, 0.95]**	4.30 (*p* = .37)	7.0	**0.66 [0.34, 0.97]**	[0.14, 1.13]	5 (60/437)	3, 6.1, 8.1, 12, 13.1
RRASOR	**0.47 [0.13, 0.81]**	**9.86 (*p* = .020)**	69.6	0.44 [-0.19, 1.06]	[-0.77, 1.64]	4 (50/190)	3, 6.1, 8.1, 11
Without outlier 8.1	0.17 [-0.24, 0.58]	3.45 (*p* = .18)	42.0	0.18 [-0.37, 0.72]	[-0.63, 0.99]	3 (36/109)	3, 6.1, 11
Any static-2002/R	**0.74 [0.26, 1.21]**	1.01 (*p* = .60)	0.0	**0.74 [0.26, 1.21]**	[0.26, 1.21]	3 (28/90)	5.1, 6.1, 7.1
Static-2002R	**0.74 [0.26, 1.21]**	1.03 (*p* = .60)	0.0	**0.74 [0.26, 1.21]**	[0.26, 1.21]	3 (28/90)	5.1, 6.1, 7.1
Mechanical	**1.13 [0.77, 1.48]**	**12.56 (*p* = .014)**	68.2	**1.09 [0.46, 1.71]**	[-0.18, 2.35]	5 (55/191)	4, 6.1, 10.1, 11, 12
Without outlier 12	**0.72 [0.29, 1.15]**	1.90 (*p* = .59)	0.0	**0.72 [0.29, 1.15]**	[0.29, 1.15]	4 (34/127)	4, 6.1, 10.1, 11
ARMIDILO-S	**1.22 [0.84, 1.60]**	**10.79 (*p* = .013)**	72.2	**1.21 [0.50, 1.93]**	[-0.15, 2.57]	4 (50/162)	4, 10.1, 11, 12
Without outliers 11 & 12	**1.09 [0.31, 1.87]**	0.66 (*p* = .42)	0.0	**1.09 [0.31, 1.87]**	[0.31, 1.87]	2 (9/54)	4, 10.1

*Notes.* Bolded value indicates statistical significance at *p* < .05. The observed number of recidivists is denoted by ‘rec’ in the *k*(rec/*N*) column. See Appendix for corresponding study authors.

**Table 3.** Prediction of Non-Sexual Violent, Any Violent, and Any Recidivism for Men With Low Intellectual Ability and Sexual Offence Histories

Prediction tools	Fixed-effect			Random-effects		*k* (rec/*N*)	Studies
	*d* [95% CI]	*Q*	*I* ^*2*^	*d* [95% CI]	*PI*		
Non-sexual violent							
Age	0.40 [−0.02, 0.82]	2.08 (*p* = .72)	0.0	0.40 [−0.015, 0.82]	[−0.015, 0.82]	5 (35/143)	5.1, 6.1, 7.1, 9.1, 10.1
Actuarial for general recidivism	0.52 [−0.0087, 1.05]	0.49 (*p* = .78)	0.0	0.52 [−0.0087, 1.05]	[−0.0087, 1.05]	3 (19/116)	5.1, 6.1, 11
Actuarial for sexual recidivism	**0.45 [0.068, 0.83]**	1.01 (*p* = .96)	0.0	**0.45 [0.068, 0.83]**	[0.068, 0.83]	6 (40/182)	5.1, 6.1, 7.1, 9.1, 10.1, 11
Any static-99/R	**0.49 [0.072, 0.90]**	0.77 (*p* = .94)	0.0	**0.49 [0.072, 0.90]**	[0.072, 0.90]	5 (35/143)	5.1, 6.1, 7.1, 9.1, 10.1
Static-99R	**0.48 [0.06, 0.89]**	0.76 (*p* = .94)	0.0	**0.48 [0.06, 0.89]**	[0.06, 0.89]	5 (35/143)	5.1, 6.1, 7.1, 9.1, 10.1
Any static-2002/R	0.30 [−0.28, 0.88]	0.51 (*p* = .78)	0.0	0.30 [−0.28, 0.88]	[−0.28, 0.88]	3 (16/90)	5.1, 6.1, 7.1
Static-2002R	0.28 [−0.30, 0.87]	0.33 (*p* = .85)	0.0	0.28 [−0.30, 0.87]	[−0.30, 0.87]	3 (16/90)	5.1, 6.1, 7.1
Mechanical	**0.98 [0.43, 1.49]**	0.41 (*p* = .81)	0.0	**0.98 [0.43, 1.49]**	[0.43, 1.49]	3 (22/111)	6.1, 10.1, 11
Any violent							
Age	**0.66 [0.34, 0.97]**	8.49 (*p* = .13)	41.1	**0.63 [0.20, 1.06]**	[−0.18, 1.44]	6 (86/186)	1.1, 5.1, 6.1, 7.1, 9.1, 10.1
Actuarial for general recidivism	**0.68 [0.28, 1.07]**	0.79 (*p* = .79)	0.0	**0.68 [0.28, 1.07]**	[0.28, 1.07]	3 (56/114)	1.1, 5.1, 6.1,
Actuarial for sexual recidivism	**0.66 [0.36, 0.95]**	3.51 (*p* = .62)	0.0	**0.66 [0.36, 0.95]**	[0.36, 0.95]	6 (85/220)	2.1, 5.1, 6.1, 7.1, 9.1, 10.1
Any static-99/R	**0.67 [0.38, 0.96]**	3.31 (*p* = .65)	0.0	**0.67 [0.38, 0.96]**	[0.38, 0.96]	6 (85/221)	2.1, 5.1, 6.1, 7.1, 9.1, 10.1
Static-99R	**0.66 [0.37, 0.95]**	3.00 (*p* = .70)	0.0	**0.66 [0.37, 0.95]**	[0.37, 0.95]	6 (85/221)	2.1, 5.1, 6.1, 7.1, 9.1, 10.1
Any static-2002/R	**0.87 [0.40, 1.33]**	1.54 (*p* = .46)	0.0	**0.87 [0.40, 1.33]**	[0.40, 1.33]	3 (38/90)	5.1, 6.1, 7.1
Static-2002R	**0.86 [0.40, 1.33]**	1.44 (*p* = .49)	0.0	**0.86 [0.40, 1.33]**	[0.40, 1.33]	3 (38/90)	5.1, 6.1, 7.1
Any recidivism							
Age	**0.76 [0.41, 1.10]**	4.88 (*p* = .30)	18.0	**0.76 [0.32, 1.20]**	[0.075, 1.45]	5 (89/156)	1.1, 5.1, 6.1, 7.1, 9.1
Actuarial for general recidivism	**0.61 [0.23, 0.98]**	1.15 (*p* = .56)	0.0	**0.61 [0.23, 0.98]**	[0.23, 0.98]	3 (67/122)	1.1, 5.1, 6.1,
Actuarial for sexual recidivism	**0.78 [0.34, 1.22]**	1.51 (*p* = .68)	0.0	**0.78 [0.34, 1.22]**	[0.34, 1.22]	4 (58/100)	5.1, 6.1, 7.1, 9.1
Any static-99/R	**0.82 [0.39, 1.26]**	1.50 (*p* = .68)	0.0	**0.82 [0.39, 1.26]**	[0.39, 1.26]	4 (58/105)	5.1, 6.1, 7.1, 9.1
Static-99R	**0.83 [0.39, 1.26]**	1.51 (*p* = .68)	0.0	**0.83 [0.39, 1.26]**	[0.39, 1.26]	4 (58/105)	5.1, 6.1, 7.1, 9.1
Any static-2002/R	**0.72 [0.26, 1.18]**	1.43 (*p* = .49)	0.0	**0.72 [0.26, 1.18]**	[0.26, 1.18]	3 (51/90)	5.1, 6.1, 7.1
Static-2002R	**0.72 [0.26, 1.18]**	1.43 (*p* = .49)	0.0	**0.72 [0.26, 1.18]**	[0.26, 1.18]	3 (51/90)	5.1, 6.1, 7.1

*Notes*. Bolded value indicates statistical significance at *p* < 0.05. PI = Prediction Interval. The observed number of recidivists is denoted by ‘rec’ in the *k*(rec/*N*) column. See Appendix for corresponding study authors.

**Figure 3.**
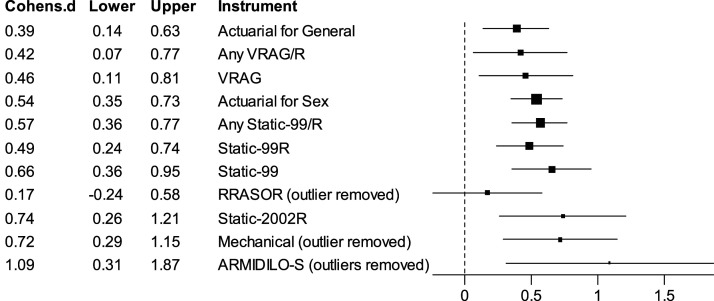
Fixed-effect averages for the relationship between risk instruments and sexual recidivism

Both fixed-effect and random-effects meta-analyses found moderate predictive accuracy for most of the risk tools across all recidivism types (see Tables 2 and 3, and Figure 3). When considering the different types of recidivism, the effect sizes were generally largest for any recidivism, followed by violent recidivism, sexual recidivism, and non-sexual violent recidivism. This pattern applied to both risk tools designed for general recidivism as well as for risk tools designed for sexual recidivism. For example, Static-99R showed a large effect for general (any) recidivism (*d* = 0.83 for fixed-effect and random-effects), moderate effects for violent recidivism (*d* = 0.66) and small to moderate effects for sexual recidivism (*d* = 0.49). These are equivalent to AUC values of 0.72, 0.68, and 0.64 for general, violent and sexual recidivism, respectively (Rice & Harris, 2005).

There was surprisingly little variability in the findings across studies. For 29 out of the 34 risk tool variables subject to meta-analysis, the between-study variability was less than would be expected by chance (*Q* < *df, I*^*2*^ = 0), the findings were identical in the fixed-effect and random-effects analysis, and the prediction intervals were identical to the confidence intervals (*T*^*2*^ estimated as zero). The reader should be cautioned, however, that the small number of studies (themselves with small samples) limits the ability of meta-analyses to detect true variability, if present.

The ARMIDILO-S provided the largest effect size for sexual recidivism (*d* = 1.22, 95% C.I. of 0.84 to 1.60 for fixed-effect, *d* = 1.21, 95% C.I. of 0.50 to 1.93 for random-effects). There was, however, significant variability (*Q* = 10.79, *p* = 0.013, *I*^*2*^ = 72.2%) between the four studies that examined this risk tool. In order to examine the influence of outliers, the two most extreme values (one high, one low) were removed. In the remaining two studies, the ARMIDILO-S was still the best predictor for sexual recidivism (*d* = 1.09, 95% C.I. of 0.31 to 1.87 for fixed-effect and random-effects; *Q* = 0.66, p = 0.42, *I*^2^ = 0), although the aggregated sample was reduced to a total of 54 cases (with 9 recidivists). Reporting results for only two studies violated our decision rules, but was preferrable to excluding only one of the outliers because the choice of either outlier would have tilted the estimates up or down. Nevertheless, this departure from the minimum number of studies limits confidence in the overall findings.

### Post Hoc Analyses

While conducting the planned analyses, we noticed that the studies that used staff reports to measure recidivism (like the ARMIDILO-S studies) appeared to have larger effect sizes than studies that relied on official criminal history records (charges, convictions, reincarcerations). Consequently, a post hoc moderator analysis was conducted with the source of sexual recidivism information (staff reports; official records) as the moderator. These analyses examined age, actuarial risk instruments for sexual recidivism, and Static-99R, each of which had enough studies to support the moderator analysis (see Table 4). ARMIDILO-S could not be included in the moderator analysis because all the studies relied on staff reports.

**Table 4.** Moderator Analysis of Sexual Recidivism Effect Sizes by Report Type (Staff vs. Official)

Predictor	Fixed-effect			Random-effects		*k* (rec/*N*)
	*d* [95% CI]	*Q*	*I* ^*2*^	*d* [95% CI]	*PI*	
Age						
Staff reports	**0.82 [0.43, 1.22]**	1.79 (*p* = .62)	0.0	**0.82 [0.43, 1.22]**	[0.43, 1.22]	4 (38/167)
Official reports	0.26 [−.040, 0.56]	1.60 (*p =* .90)	0.0	0.26 [−.040, 0.56]	[−.040, 0.56]	6 (60/271)
Q_between_	**4.97 (*p* = .026)**			**4.97** (*p* = .026)		
Actuarial for sexual recidivism						
Staff reports	**0.68 [0.38, 0.99]**	5.88 (*p* = .21)	31.9	**0.70 [0.29, 1.1]**	[0.015, 1.38]	5 (64/243)
Official reports	**0.44 [0.19, 0.69]**	3.71 (*p* = .81)	0.0	**0.44 [0.19, 0.69]**	[0.19, 0.69]	8 (84/532)
Q_between_	= 1.46 (*p* = .23)			= 1.09 (*p* = .30)		
Static-99R						
Staff reports	**0.91 [0.15, 1.68]**	0.22 (*p* = .64)	0.0	**0.91 [0.15, 1.68]**	[0.15, 1.68]	2 (9/54)
Official reports	**0.44 [0.18, 0.71]**	3.64 (*p* = .73)	0.0	**0.44 [0.18, 0.71]**	[0.18, 0.71]	7 (76/491)
Q_between_	= 1.29 (*p* = .26)			= 1.29 (*p* = .26)		

*Notes*. Official reports include arrests/charges or convictions. Bolded value indicates statistical significance at *p* < 0.05. PI = Prediction Interval. The observed number of recidivists is denoted by ‘rec’ in the *k*(rec/*N*) column.

The effect size for age predicting sexual recidivism was large in studies that measured recidivism by staff reports (*d* = 0.82) and small in studies that relied on official reports (*d* = 0.26; *Q*_*between*_ = 4.97, *p* = .026). The same pattern was observed for sexual recidivism actuarial instruments (0.70 versus 0.44) and Static-99R in particular (0.91 versus 0.44). Readers should be cautioned, however, that the latter comparisons (i.e., actuarial instruments and Static-99R) were not statistically significant, and there were only two Static-99R studies that used staff reports to measure recidivism.

## Discussion

Empirically validated risk instruments can increase objectivity and decrease the error and bias of unstructured clinical judgement often decried in the literature (Monahan, 2008; Viljoen et al., 2025). All applications of scientific evidence, however, involve inferences from patterns in group data to the specific characteristics of the case-at-hand. Inferences from risk instruments are easiest to support when the case closely resembles the populations upon which the risk instrument was developed and validated, which are mostly White, WEIRD (Henrich, 2024), and neurotypical. Consequently, evaluators should approach risk assessments of men with clinically significant low intellectual ability (i.e., developmental disorders) with extra caution and care.

The current results provide some confidence that the existing risk instruments for crime and violence work as intended for men with low intellectual ability and a history of sexual offending. Most measures demonstrated moderate predictive validity (discrimination), with the largest effect sizes for any recidivism, followed by violent recidivism, sexual recidivism, and non-sexual violent recidivism. The same pattern applied to risk tools for general crime as well as for risk tools explicitly designed to assess sexual recidivism. For example, the *d* value for Static-99R predicting any recidivism was 0.83 whereas it was 0.49 for sexual recidivism. For sexual recidivism, the instrument with the largest effect was the ARMIDILO-S (*d* > 1.0) and the instrument with smallest effect was for the RRASOR (*d* = 0.17).

We are not sure why general (any) recidivism appeared easier to predict than sexual recidivism. The confidence intervals were wide and overlapping; consequently, the manifest pattern may be simply due to chance. It is possible, however, that general recidivism is more accurately recorded than sexual recidivism. Low base rates make for unreliable recidivism analyses (Barbaree, 1997). Because base rates for any recidivism are (necessarily) higher than for sexual recidivism, any recidivism may have been easier to detect. It could also be that early non-sexual recidivism activate system responses (e.g., close supervision) that inhibits the likelihood of subsequent sexual recidivism. As pointed out by our reviewers, this is the ever-present problem of competing hazards. Initial failures of one type can influence the likelihood of another type (e.g., dying by cancer rules out subsequent death by heart disease, and vice versa).

One of the notable incidental findings was the larger effects found when recidivism was measured by staff reports compared to official records (e.g., charges, convictions). This effect cannot be attributed to contamination between the outcome measure (staff reports of sexual transgressions) and the predictor variables (many of which are informed by staff ratings) because the same pattern was observed for age, which should be immune to this type of bias. Instead, staff reports appear to be a more reliable indicator of criminal behaviour than official records for the men with low intellectual ability. Given that a large number of persons with clinically significant low intellectual ability and sexual behaviour problems are found in “care” settings (e.g., group homes, state-run hospitals, private facilities), they are subject to high levels of observation and risk management. When allowed in the community (if at all), they are often subject to eyes-on, arms-length supervision, sometimes at a 2:1 supervisor to individual level. For the higher risk cases, such high levels of supervision are routinely in place even inside their place of residence. Given that many offences committed by persons with low intellectual ability are dealt with in-house and not officially reported (Taylor & Lindsay, 2018), it comes as no surprise that staff reports of new sexual crimes (32.2%) outstrip those of official reports (17.8%) in our samples.

In response to reviewer feedback, we conducted further post hoc analyses comparing the predictive accuracy of age and actuarial risk tools in community versus institutional settings. There were no significant differences (see supplemental materials: https://osf.io/7vr43/overview). It is quite likely, however, that many of the settings we coded as community settings provided levels of daily supervision that equalled the supervision in institutional settings.

Based on an average 7-year follow-up, the sexual recidivism rates of the current samples were much higher (19.5% official sexual recidivism rates) than is found for typical, routine correctional samples (e.g., average risk, routine samples: 3.2-6.5% expected sexual recidivism rates after 5-years in Helmus et al., 2021). This elevated rate likely reflects the nature of the included samples, which were drawn primarily from specialized treatment settings rather than routine correctional populations. Although the samples were above average risk on the standardized risk tools, we were unable to examine the extent to which the observed recidivism rates aligned with the expected rates provided by the norms of the actuarial risk tools. Prior research has shown that base rates can vary substantially depending on sample characteristics and follow-up procedures (Helmus, Hanson, et al., 2012), with specialized or high-risk groups often yielding higher recidivism rates. Consequently, the elevated rates reported here may not generalize to broader groups of men with low intellectual ability.

Age as single variable showed surprisingly large effect sizes, which were comparable to those of the structured risk tools (the *d* values for age were 0.46, 0.66, and 0.76 for sexual, violent, and any recidivism, respectively). Hanson and Bussière’s (1998) meta-analysis found a much smaller effect of age on sexual recidivism (*d* = .26) and any recidivism (*d* = .32) in mixed samples of men with a history of sexual offending. There are several potential explanations. As younger men with intellectual deficits move into adulthood, their offending behaviours may be taken more seriously, resulting in transition from their family home to a more specialized group home setting or institution. This change may prevent reoffending in older men with low intellectual ability due to increased supervision, greater access to behavioural interventions, and the overall transition to a more stable, supportive environment. Once a controlled environment is established, they can be safely managed until their risk is reduced to very low levels by natural aging processes common to us all (e.g., decreased sexual drive, illness, increased dependence on others). Such special circumstances could contribute to a large protective effect of aging in men with low intellectual ability.

It is also possible that men with developmental delays take a longer time to establish sexual self-regulation and learn acceptable sexual behaviours. The same developmental challenges associated with sexual crime by adolescents of average intellectual ability may not resolve until well into the adult years for men with low intellectual ability. Even if they maintain a low ceiling on their intellectual functioning, they may still learn that “crime doesn’t pay” – it just takes more time. The ongoing, if delayed, life learning process among men with disorders of intellectual development may increase the importance of aging during adulthood as a protective factor for crime.

A related explanation is that, compared to mixed samples, sexual offending by men with low intellectual ability is more strongly connected to general self-regulation deficits and less connected to sex crime specific factors (e.g., atypical sexual interests, sexualized coping). This variation on the counterfeit deviance hypothesis would help explain the high predictive accuracy of sexual recidivism risk tools for general recidivism and suggest that the strong link between age and sexual recidivism is mediated by general self-regulation deficits.

It is difficult to determine how much of the strong predictive accuracy of the ARMIDILO-S (*d* = 1.09) should be attributed to special features of the instrument and how much to better recidivism information. When staff reports were used as the outcome measure, Static-99R was not far behind (*d* = 0.91). There are, however, distinctive features of the ARMIDILO-S that could make it particularly effective with men with low intellectual ability. Of all the risk instruments reviewed, it was the only one explicitly designed for this population. Consequently, its variables and rating system were tailored to the distinctive characteristics of men with low intellectual ability and a history of sexual offending. Importantly, it requires staff engagement to score and to carefully consider the men’s living conditions and supervision/case management frameworks. Another distinctive feature is each item is evaluated as both a risk factor and a protective factor. Even when protective factors do not address new domains, directing evaluators to consider “good” functioning (along with the bad) can improve the predictive accuracy of violent and sexual recidivism risk assessments (e.g., Burghart et al., 2023; Thornton, Willis, & Kelley, 2024).

### Limitations and Future Directions

Although the research has increased since Hanson et al.’s (2013) previous meta-analysis, the number of studies remains small, and the studies themselves had relatively few cases. Consequently, the confidence intervals often spanned the range from small (*d* < .30) to large (*d >* 0.80). If the true effect size is actually small, the risk instruments would have limited practical utility. Nevertheless, the average values provide defensible estimates of the true effects, which were moderate to large for most measures and most outcomes. To address this lack of large studies, future studies should prioritize larger sample sizes and multi-site collaborations to narrow confidence intervals and improve the precision of effect size estimates. The small sample sizes similarly prevented analyses of subgroups within the subgroup of men with low intellectual ability. For the studies reviewed, the majority of the sexual offending involved the victimization of unrelated children. Consequently, we have less confidence that our results would generalize to men whose offending history exclusively involved sexual offences against adults, or non-contact sexual offenses.

We only examined predictive accuracy in terms of discrimination (how well the instruments differentiated between recidivists and non-recidivists), not calibration (the match between the expected and observed recidivism rates; see Hanson, 2022, Part IV). Even if risk instruments are able to accurately rank order the men from lowest to highest risk, the recidivism rates norms from the actuarial measures may be too high or too low when applied with men with low intellectual ability and a history of sexual offending. Future research should investigate the calibration of these tools with this population to ensure that their normative risk estimates are valid and appropriate for applied settings.

Inconsistencies in the source materials limit confidence in the findings of meta-analyses. In this study, perhaps the most significant inconsistency concerned the population studied. Although all studies identified men with low intellectual ability, their selection criteria varied. Some used formal diagnoses, some privileged standardized testing, some included men with Borderline Intellectual Functioning, and some studies did not specify how low intellectual ability was ascertained. Follow-up time varied from 5 months to 20 years. The variation in the outcome definitions (e.g., whether they used staff report or official sources of recidivism) restricted our ability to determine whether observed variation in predictive accuracy reflects true differences in risk tools or methodological artifacts. Future research should consider using multiple sources of outcome data (staff and official) to increase reliability and validity.

Relatedly, the intrinsically hidden nature of sexual offending presents a challenge to sexual recidivism research. This concern, however, may be mitigated to some extent when studying men with low intellectual ability, whose daily routines and interpersonal contacts, including potential victims, are often monitored closely by support staff. As a result, staff are more likely to observe or learn about transgressive behaviour that may otherwise go unnoticed. Future researchers should therefore privilege staff reports when conducting recidivism research with this population. If the differences in predictive accuracy between staff reports and official reports are to be believed, it is likely that the predictive accuracy of risk instruments used with other populations (without disorders of intellectual development) is actually higher than commonly reported in the literature because the risk tools themselves can be “better measures of criminal behaviour than any official re-arrest or reconviction record” (Vrieze & Grove, 2010, p. 388). It would be valuable to systematically follow staff-reported incidents to examine which cases result in official charges and which are addressed internally. This line of research could help clarify the boundary between criminal and non-criminal management in this population and improve the interpretation of official recidivism outcomes.

Another promising program of research would examine the construct validity of recidivism risk indicators for men with low intellectual ability. For men with a history of sexual offending, most of the risk indicators can be grouped into the broad categories of general criminal and sexual criminality, with a less stable third factor related to young age and non-sexual violence (Brouillette-Alarie et al., 2016). Would similar factor structure and item loadings be found in men with low intellectual ability? In other samples, having male victims loads on the sexual criminality factor and is a reliable indicator of pedophilia (Seto et al., 2017). This may or may not be the case for men living in male-only facilities where victim choice could be influenced by proximity or opportunity rather than sexual preference. Further work is needed to validate the interpretation of key indicators in this population to ensure accurate assessment and treatment planning.

Further research is also needed on counterfeit deviance – the tendency to attribute deviant intent to acts that have other, nondeviant explanations (Griffiths et al., 2013; Hingsburger et al., 1991). For example, masturbating in a common area home may not be motivated by a paraphilia for exhibitionism; instead, it may be related to a lack of privacy, attention-seeking, task avoidance (i.e., getting in trouble may exempt the individual from doing chores), or a simple inability to cognitively appreciate the wrongness of the behaviour or the location in which it is done. Understanding the *etiology* of the transgression is an important step to developing an effective intervention strategy.

Another direction for future research concerns the extent to which the use of risk tools promote better outcomes. In practice, evaluators using structured professional judgment (SPJ) tools, such as the ARMIDILO-S, summarize their risk assessment into broad categories of low, moderate and high, which communicate recommendations for the amount of support and supervision needed. We did not find enough studies of SPJ ratings to meta-analyze their predictive accuracy. Future reviews should consider how the use of risk tools influence the actual services provided, and the extent to which services that are informed by risk tools leads to improved outcomes.

### Implications for Practice

The current results support the use of recidivism risk tools with men with low intellectual ability and a history of sexual offending. Although prudent evaluators are particularly cautious when applying risk instruments to understudied groups, the risk scores from the existing tools provide a plausible basis for differentiating men with low intellectual ability into lower and higher risk groups. For sexual recidivism, there is sufficient evidence to recommend Static-99R, Static-2002R, and ARMIDILO-S (and not the RRASOR) for this purpose. Our recommendation to use Static-99R rather than Static-99 was based on (a) a large corpus of research supporting Static-99R (Helmus et al., 2022), and (b) Static-99 is no longer supported by its authors (Thornton & Hanson, 2016). Although the meta-analytic average for Static-99 was (non-significantly) higher than for Static-99R, we attribute this to differences in the studies included and not to the superiority of Static-99 over Static-99R for this population. In the two studies that directly compared Static-99 and Static-99R, the effect sizes were very similar for both measures (*d* = 1.06 for Static-99, 1.03 for Static-99R, Hanson et al., 2024 [6.1]; *d* = 0.19 for Static-99; 0.16 for Static-99R, Callahan et al., 2024 [13.1]).

ARMIDILO-S would be the go-to measure for comprehensive evaluations that inform case management plans. Given that the ARMIDILO-S and the STATIC instruments are based on quite different types of information, there could be benefits from using the ARMIDILO-S in combination with one of the STATIC measures, which parallels the recommendations of the ARMIDILO-S Scoring Manual (Boer et al., 2013). To our knowledge, there are no validated, mechanical methods for combining these risk tools (a gap that represents a valuable research opportunity). Consequently, professional judgement is needed to integrate the information from the different instruments into an overall risk assessment and case management plan.

The use of static actuarial measures with the men with low intellectual ability is not without practical challenges. Given that young age was found to be a much stronger predictor of recidivism in this population than in other populations, the age thresholds and weights in existing measures are unlikely to be optimized for men with significant cognitive delays. Furthermore, age scoring of Static-99R and Static-2002R requires a date of release. According to the Static-99R scoring manual (Phenix et al., 2017), men are not considered “released” if they are living in an institution or a treatment facility on an involuntary basis, or living in the community under severe restrictions. Such conditions apply to most men with low intellectual ability and a history of sexual offending and even those individuals who are housed on a “voluntarily” basis who are often subject to considerably higher scrutiny due to their intellectual and other challenges. Consequently, we recommend that evaluators using Static-99R and Static-2002R score them in accordance with their scoring manuals, and consider current age as an additional, external risk factor for men living in secure settings, even if there is no foreseeable release date.

### Conclusion

Persons with low intellectual ability are often marginalized in society, and this marginalization may be even greater for those who commit sexual offences. The results from the studies presented in this meta-analysis indicate that men with low intellectual ability who have committed sex offences vary in risk for recidivism; some are more likely to commit an offence again, whereas others are much less likely to reoffend. The findings of this meta-analysis suggest that evaluators or case managers involved with this specific sub-group can use existing methodologies to help make decisions concerning matching risk levels to public protection measures and rehabilitation services. By doing so, people working with this population can promote the safety and well-being of all.

## Appendix

**Table A1.** Legend for Tables: Study ID Numbers

Study number	Authors	Study number	Authors
1.1	Curry (2016) ^a^	8.1	Tough (2001)^a^
1.2	Fedoroff et al. (2016)	8.2	Tough (2001)
2.1	Stephens et al. (2018)	8.3	Harris and Tough (2004)
2.2	Stephens et al. (2018)^a^	9.1	Baudin et al. (2021) ^a^
3	Wilcox et al. (2009)	9.2	Baudin et al. (2021)
4	Sindall (2012)	10.1	Pouls and Jeandarme (2023)^a^
5.1	Hanson et al. (2024)^a^	10.2	Pouls and Jeandarme (2022)
5.2	Hanson and Harris (2000)	10.3	Pouls and Jeandarme (2023)
5.3	Hanson et al. (2024)	11	Blacker et al. (2011)
5.4	Blais et al. (2024)	12	Lofthouse et al. (2013)
5.5	Aelick et al. (2020)	13.1	Callahan et al. (2024)^b^
6.1	Hanson et al. (2024)^a^	13.2	Callahan et al. (2024)
6.2	Hanson et al. (2007)	14.1	Olver et al. (2007)^a^
6.3	Hanson et al. (2015)	14.2	Sowden and Olver (2017)
6.4	Hanson et al. (2013)	15.1	Delforterie et al. (2023)^a^
7.1	McGrath et al. (2007)^a^	15.2	Delforterie et al. (2023)
7.2	McGrath et al. (2007)		
7.3	McGrath et al. (2012)		

^a^Unpublished raw data.

^b^Supplemental materials to published article.
